# Common Features of Selection Processes of Health System Performance Indicators in Primary Healthcare: A Systematic Review

**DOI:** 10.34172/ijhpm.2022.6239

**Published:** 2022-03-17

**Authors:** Nicole Rendell, Alexander Rosewell, Kamalini Lokuge, Emma Field

**Affiliations:** ^1^National Centre for Epidemiology and Population Health, Australian National University, Canberra, ACT, Australia.; ^2^School of Public Health and Community Medicine, University of New South Wales, Sydney, NSW, Australia.; ^3^Menzies School of Health Research, Brisbane, QLD, Australia.

**Keywords:** Performance Indicators, Primary Healthcare, Selection, Development

## Abstract

**Background:** Health system performance indicators are widely used to assess primary healthcare (PHC) performance. Despite the numerous tools and some convergence on indicator criteria, there is not a clear understanding of the common features of indicator selection processes. We aimed to review the literature to identify papers that document indicator selection processes for health system performance indicators in PHC.

**Methods:** We searched the online databases Scopus, Medline, and CINAHL, as well as the grey literature, without time restrictions, initially on July 31, 2019 followed by an update November 13, 2020. Empirical studies or reports were included if they described the selection of health system performance indicators or frameworks, that included PHC indicators. A combination of the process focussed research question and qualitative analysis meant a quality appraisal tool or assessment of bias could not meaningfully be applied to assess individual studies. We undertook an inductive analysis based on potential indicator selection processes criteria, drawn from health system performance indicator appraisal tools reported in the literature.

**Results:** We identified 16 503 records of which 28 were included in the review. Most studies used a descriptive case study design. We found no consistent variations between indicator selection processes of health systems of high income and low- or lower-middle income countries. Identified common features of selection processes for indicators in PHC include literature review or adaption of an existing framework as an initial step; a consensus building process with stakeholders; structuring indicators into categories; and indicator criteria focusing on validity and feasibility. The evidence around field testing with utility and consideration of reporting burden was less clear.

**Conclusion:** Our findings highlight several characteristics of health system indicator selection processes. These features provide the groundwork to better understand how to value indicator selection processes in PHC.

## Introduction

 Health system performance indicators are used to understand how well a health system is functioning and to what level it is meeting the needs of the population it has been designed to serve. This is often specific to the country context, which uses the country expectations of their health system, to develop such indicators. Regardless of context, indicators must be developed in a way that ensures any monitoring, evaluation or review activities, accurately reflect the health priorities, local health needs and expectations regarding equitable quality of care. The coronavirus disease 2019 (COVID-19) pandemic has further highlighted the value of timely access to health system performance intelligence and the central role of primary healthcare (PHC). However, striking the balance between detailed information from numerous indicators and a smaller number of clearly communicated indicators that can be reasonably collected, is an ongoing challenge.^1,2^ Both extremes can lead to a situation where there is a weak correlation between the performance indicators and the performance itself, negating the value of indicators and performance measurement.^1^

 There are many studies in the literature on health system performance assessment. Some studies have tried to align health system performance indicators between countries to draw comparisons.^3-8^ However often international comparability is only possible across core health system components, such as those defined by the World Health Organization (WHO) to align with their six building blocks framework.^3,5,7-9^ There are also instruments available in the literature that have been developed to analyse a given set of quality indicators in the healthcare system after they have been created, such as QUALIFY and the Appraisal of Indicators through Research and Evaluation (AIRE).^10,11^ However, in the context of selecting health system performance indicators, there is little consensus on the approach to ensure the indicators are fit for use. In this context, we apply the concept described by Barbazza et al where fit for purpose and fit for use are both key constructs of actionable indicators. Specifically, fit for use is defined as “*getting the right information into the right hands at the right time*” (p. 2).^12^ We extend this definition to also consider reporting burden and other implementation factors.

 While there is some literature available that describes the selection of health system performance indicators or a broader performance framework in a given context,^4,13-18^ there appears to be no comprehensive review of the processes used to select them, and how they compare.^19,20^ One recent systematic review considered content validity of indicator sets specifically across the full spectrum of healthcare settings. Procedural criteria formed only part of the review’s findings and included consideration of assessment purpose, develop/use conceptual framework, stakeholder involvement and transparency of the development process.^21^ We ascertain that a set of health system performance indicators could be considered successful if they were fit for use. However, in the first instance, we must understand what the current approaches to selection of health system performance indicators are and in what ways they vary. We aimed to review the literature to identify papers that document indicator selection processes for health system performance indicators in PHC.

 For the purposes of this research, we will focus on health system performance indicators used to measure performance in PHC. Indicators at this level of the health system focus on local service delivery ranging from preventive services such as vaccinations, to ongoing management of non-communicable diseases.^22^ PHC has long been considered integral to health system functioning ^23-27^ and there are many health system frameworks and indicators available that have been devised to measure and assess PHC specifically.^28-30^ These include Primary Care Assessment Tools^30^ and the Primary Health Care Performance Initiative^29^ in addition to broader health system frameworks such as the WHO Health System Building Blocks ^9^ and the Sustainable Development Goals.^31^ Despite a range of standardised PHC frameworks and indicators to choose from, each with tools and resources available to support countries in implementing them, evidence suggests they are not consistently implemented.^8,20,32-34^

## Methods

 This systematic review was completed in accordance with the Preferred Reporting Items for Systematic Reviews and Meta-Analyses (PRISMA) guidelines.^35^

###  Search Strategy and Selection Criteria 

 We searched three online databases (Scopus, Medline and CINAHL), without time restrictions, and the grey literature (using the global search function on the Google platform in privacy mode, first 300 citations) on July 31, 2019. An updated database search followed on November 13, 2020. The searches for the online databases used the following syntax (see Supplementary file 1 for full details):

 (“health system?” OR “health care” OR “primary health*” OR “primary care”) AND (“performance indicator?” OR “quality indicator?” OR “framework?”) AND (development OR prioriti?ation OR selection) AND NOT (acute OR hospital).

 The grey literature search simply included a global search of the phrase: “development of health system performance indicators in primary care.” The databases were selected based on their reputation for content on health systems. The approach to the grey literature was adopted after testing a range of terms and search conditions, which found that a simplified global search was most effective in returning relevant results. The reference lists of all included studies were also searched for further eligible studies.

 Studies were eligible for inclusion if the full text was available in English and they were empirical studies or reports in the grey literature that described the selection of health system performance indicators or frameworks, that included PHC indicators. These included clinical indicator series covering more than one disease and are used with the goal of understanding PHC performance. Indicator selection processes included any indicator or indicator set, that was identified for implementation and ongoing PHC management in a real world setting. The care setting was considered in scope, if it aligned with the definition of PHC outlined by WHO^22^ and no referral was required by an individual to seek the services. Although we did not record exclusions from failing the PHC criterion when completing the title and abstract screening. It was also necessary for inclusion, that the indicators were field tested, piloted or implemented (field testing). This was interpreted to include revisions of an existing indicator set and those of well-established organisations known to the authors with a clear trajectory for implementation, of indicators yet to be clearly implemented. This criterion was to ensure practical considerations of implementation were captured by the selected studies.

 Empirical studies that reported only on indicators related to hospitals or acute settings; and developed and/or applied only a survey design without consideration for selection of PHC indicators were excluded. Studies were also excluded if they were secondary sources (for example, narrative reviews and systematic reviews); the indicators were specific to a single condition, due to our focus on health system performance; or based on a theoretical discussion on health system performance assessment including proposed indicators or frameworks that had not been field tested.

 Two reviewers independently screened all titles, abstracts and full text articles according to the inclusion and exclusion criteria. Differences were resolved by consensus. Where the selection process of the same framework was reported across two papers, only the most comprehensive or more recent publication was selected for full text review and inclusion.

###  Data Extraction

 For included studies or reports, we extracted information on the country context and key features of indicator selection including scale (international, national or subnational level indicators); type of indicator(s) (ie, subject); whether it was an original framework or revision; the key steps taken to develop the relevant indicator(s); consideration of existing frameworks and/other global reporting requirements; consideration of causal chain (for example, use of a logic model); stakeholders consulted; data quality and validity; data availability, reliability and coherence with existing systems; reporting burden/resources relative to the context; and ongoing review of indicators. These criteria for data extraction were informed by health system indicator appraisal tools reported in the literature^10,11,36^ and then agreed by all authors.

###  Quality Assessment

 The premise of this work is to summarise common approaches to understand possible factors in reported health system performance indicator selection processes that could potentially be used to assess the value of one indicator selection process over another. This is laying the groundwork for development of a quality assessment tool of indicator selection *processes*. To determine relevant criteria for data extraction, we used published indicator appraisal tools that assessed the quality of health system performance indicators themselves, ie, *outcomes*, to inform the structure of our dataset on *processes*.^10,11,36^ This infers that such criteria applied to indicators, is a valid starting point for understanding different aspects of processes used to select them. The criteria are outlined in the previous section, under *Data Extraction*, and were agreed to by consensus among authors. Therefore, the quality assessment of included papers formed part of the data extraction process used to answer our research question and was informed by categories for assessment of indicator quality which were agreed by all authors. For this reason, application of a quality appraisal tool or assessment of the risk of bias were not appropriate.

###  Data Analysis

 Following data extraction, indicator selection processes were compared and contrasted against the criteria drawn from indicator appraisal tools,^10,11,36^ their country context, emerging themes and patterns of interest. The analysis was inductive by design and allowed for key themes to emerge from the qualitative data extracted.

## Results

###  Search Results

 Our search identified 16 503 records of which 16 404 were excluded after screening titles and abstracts. After assessing the full text of 99 articles, we excluded 71 because there was insufficient information for data extraction (n = 20), not an empirical study (n = 12), a more comprehensive paper was available for inclusion (n = 10), the care setting was not PHC (n = 9), the indicators were specific for a single condition (n = 8), the testing criteria was not met (n = 7) or the full text was not available in English (n = 5). In total 28 studies and reports were included for analysis (see Figure).

**Figure:**
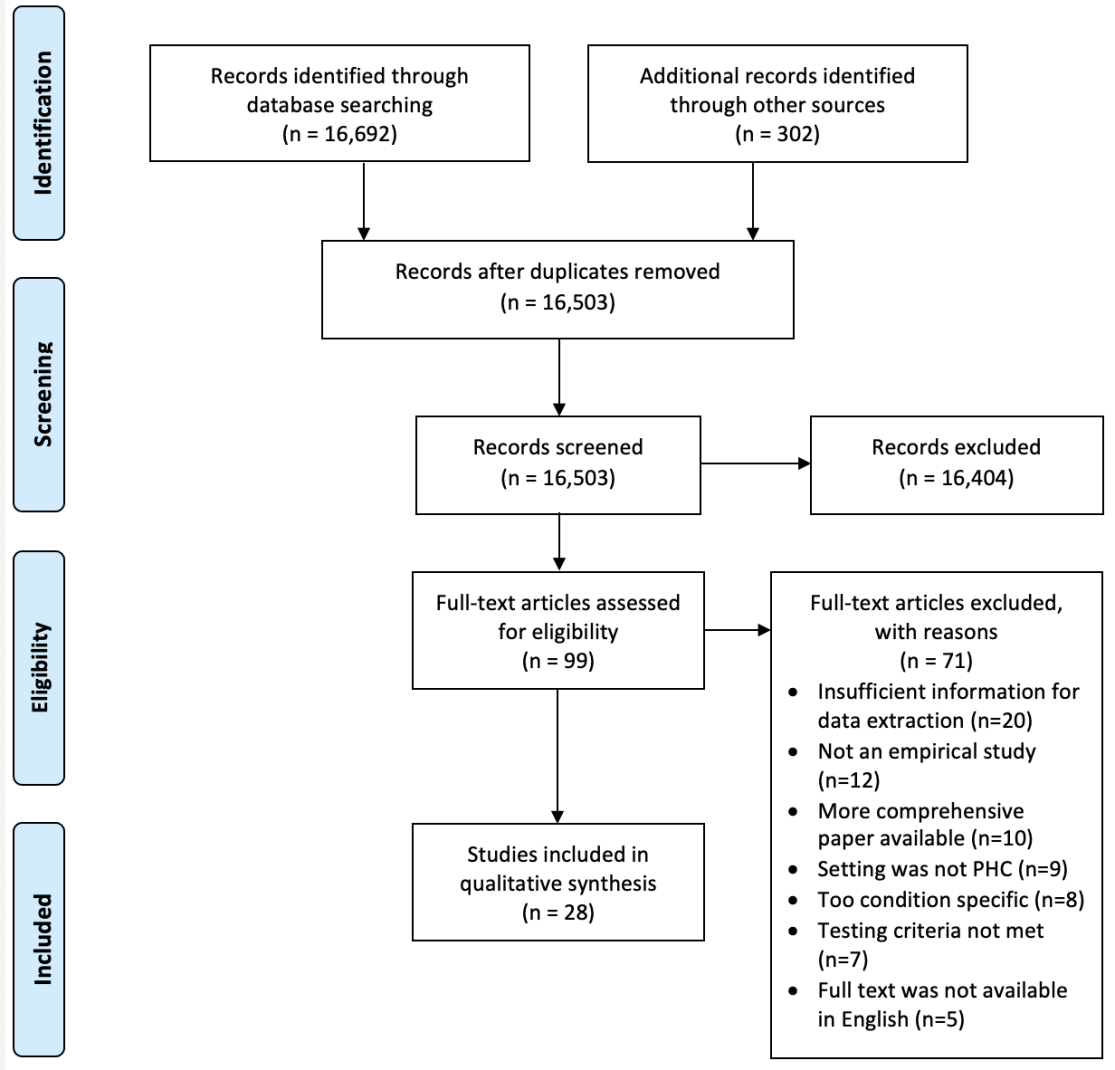
Flow Diagram of Screening Process.

###  Study Characteristics

 The characteristics of the included studies are summarised in Table 1. The included studies were classified according to the World Bank Country and Lending Groups^37^ at the time of their publication. They were predominantly from high income countries (n = 23) of which 15 were Europe centric,^38-52^ three each from the United States^53-55^ and Canada^56-58^ and one each from Australia^59^ and New Zealand.^60^ The few papers from upper-middle (n = 1)^61^ or low- and middle-income countries (n = 4), were set in Asia and Africa including China, Nepal, Kenya and India.^62-65^ The selection criteria meant that most of the included papers centred around a descriptive case study methodology (n = 23).^39-44,46,48-56,58-63,65^ The other papers were an exploratory case study (n = 1)^57^ or cross sectional studies (n = 4).^38,45,47,64^ Half of the included papers were published in last six years from 2015 (n = 14),^38-40,42,45,49-52,54,56,62,63,65^ with the oldest included paper published in 1998.^53^ All themes described below form the results from the analysis.

**Table 1:** Characteristics of Included Studies

**Reference**	**Care Setting**	Study Type*	**Scale, Type of Indicator(s)**	**Name of Framework Developed or Project **
Aller et al, 2015^38^	Three healthcare areas of the Catalan health system, Spain	Cross-sectional	Sub-national, coordination of care	NA
AIHW, 2009^59^	Healthcare services, Australia	Government report	National level, safety and quality	National Indicators of Safety and Quality in Healthcare
Barbazza et al, 2019^39^	Primary care practices, Europe	Case study - descriptive	International, PHC performance	PHC-IMPACT
Barrett et al, 1998^53^	Two community mental health agencies in Colorado, United States	Case study - descriptive	Sub-national, mental health services performance	NA
Blozik et al, 2018^40^	Swiss mandatory basic health insurance (Helsana group), Switzerland	Case study - descriptive	National, quality of care of ambulatory services	NA
Campbell et al, 2011^41^	Health services, United Kingdom	Case study - descriptive	National, clinical and organisational quality	QOF
Carinci et al, 2015^42^	OECD countries	Case study - descriptive	International, healthcare quality	Healthcare Quality Indicators project
Claessen et al, 2011^43^	Palliative care services, the Netherlands	Case study - descriptive	National, palliative care	NA
Coma et al, 2013^44^	PHC professionals in Catalonia, Spain	Case-study - descriptive	Sub-national, PHC quality	EQA
Cookson et al, 2016^45^	Small-area level, England	Time-series cross-sectional	Sub-national, health equity	NA
De Bie et al, 2011^46^	Community pharmacies, Netherlands	Case study - descriptive	National, community pharmacy care	NA
Engels et al, 2006^47^	General practices in selected European countries - Austria, Belgium, France, Germany, Israel, The Netherlands, Slovenia, Switzerland and the United Kingdom	Cross-sectional	International, management of primary care practices in Europe	EPA project
Gribben et al, 2002^60^	First Health network of general practices, New Zealand	Case study - descriptive	Sub-national, PHC quality	NA
Herndon et al, 2015^54^	Pediatric oral healthcare in the United States	Case study - descriptive	National, pediatric oral care	NA
Hutchison et al, 2020^56^	PHC in Ontario, Canada	Case study - descriptive	Sub-national, PHC performance	PCPM
Katz et al, 2006^57^	Family practices in Manitoba, Canada	Case study - exploratory	Sub-national, PHC quality	NA
Leemans et al, 2013^48^	Palliative care in Flanders, Belgium	Case study (protocol) - descriptive	Sub-national, Palliative care	Q-PAC
Nambiar et al, 2020^62^	PHC facilities in Kerala, India	Case study - descriptive	Subnational, PHC performance	NA
Parker et al, 2015^49^	Patient safety in primary care organisations, Europe	Case study - descriptive	International, patient safety in PHC	LINNEAUS collaboration
Prytherch et al, 2017^63^	Antenatal Care, Postnatal, Family Planning and Maternity services, Kenya	Case study - descriptive	National, PHC quality	KQMH
Reedy et al, 2005^55^	Santa Clara County public health services, United States	Case study - descriptive	Sub-national, patient safety in PHC	NA
Riain et al, 2015^50^	General Practices in Ireland	Case study - descriptive	National, PHC quality	GP-IQ
Rushforth et al, 2015^51^	Primary care practices, United Kingdom	Case study - descriptive	National, PHC quality	NA
Sarriot et al, 2009^64^	Health districts, Nepal	Cross-sectional	Sub-national, health sector aid investments	Nepal Specific Sustainability Framework
Stanciu et al, 2020^52^	Primary care clusters, Wales	Non-experimental mixed methods	Subnational, PHC performance	PCCMA
Terner et al, 2013^58^	PHC, Canada	Case study - descriptive	National, PHC performance	NA
Veillard et al, 2017^65^	PHC systems in 135 low- and middle-income countries	Case study - descriptive	International, PHC quality	PHCPI
Wong et al, 2010^61^	PHC, China	Case study - descriptive	Sub-national, PHC performance	China results- based Logic Model for CHS

Abbreviations: NA, Not Applicable; PHC-IMPACT, Primary Healthcare Impact, Performance and Capacity Tool; AIHW, Australian Institute of Health and Welfare; QOF, Quality and Outcomes Framework; OECD, Organisation for Economic Co-operation and Development; EQA, the Catalan acronym for Estàndard de Qualitat Assistencial; EPA, European Practice Assessment; PCPM, Primary Care Performance Measurement; Q-PAC, Quality Indicators for Palliative Care; KQMH, Kenya Quality Assurance Model for Health; GP-IQ, General Practice Indicators of Quality; PCCMA, Primary Care Clusters Multidimensional Assessment; PHCPI, Primary Healthcare Performance Initiative; CHS, Community Health Facilities and Stations.

###  Country Context

 All indicator selection processes that were set in low- and low-middle income countries (n = 4)^62-65^ were published relatively recently, with the earliest published in 2009^64^ and the other three papers published within the last four years.^62,63,65^ There were no other consistent characteristics noted among papers from low- and low-middle income countries. However, all papers that were included as revisions (n = 4) were set in high income countries,^41,42,56,58^ two of which were set in Canada, one focusing on subnational indicators in Ontario.^56^

###  Key Steps

 The included papers highlighted common steps taken to develop health system performance indicators in PHC (see Table 2). Most of the indicator selection processes began with a review of the literature and other policy documents. Some of these adopted a stricter methodology opting for a rigorous systematic review (n = 5)^38,39,43,48,52^ as an initial step, while the others adopted a more flexible approach to reviewing the literature and other publicly available documents (n = 13).^42,46,49,50,54,55,57-60,62,63,65^ All those that did not explicitly use a literature review as part of their indicator selection process, relied heavily on adapting an existing framework into the relevant context (n = 10).^40,41,44,45,47,51,53,56,61,64^

**Table 2:** Number of Included Papers by Indicator Selection Feature

	**High-Income**	**Upper-Middle Income**		**Low- and Lower-Middle Income **
Country context	23^38-60^	1^61^		4^62-65^
	**Systematic Review**	**Literature Review**		**No Review – Relied on Existing Framework**
Review of existing publications	5^38,39,43,48,52^	13^42,46,49,50,54,55,57-60,62,63,65^		10^40,41,44,45,47,51,53,56,61,64^
	**Delphi**	**RAND/UCLA Appropriateness **	**No Formal Methodology Applied**	**None Reported**
Consensus process	7^42,44,47,49,50,62,65^	5^41,48,51,54,63^	14^38-40,43,45,46,52,53,56-59,61,64^	2^55,60^
Engagement with patients	2^47,50^	3^48,51,54^	4^40,43,45,56^	
Field Testing	5^47,49,50,62,65^	5^41,48,51,54,63^	5^39,40,43,46,52^	1^60^
	**Categories/Dimensions**	**Donabedian**	**Logic Model**	**Single Composite Indicator**
Structure of indicators	22^38,40-43,45-51,53-60,63,64^	2^39,52^	3^61,62,65^	1^44^
	**Validity **	**Feasibility **	**Burden of Reporting **	**Ongoing Review **
Indicator criteria	All	All	13^39,41-44,48,50,51,53,56,59,62,64^	6^41,42,47,56,59,62^

Total Included papers = 28.

 All of the included indicator selection processes incorporated a consensus process with context specific stakeholders to reach the final set of indicators, with two exceptions.^55,60^ In just under half of the selected papers, consensus building was achieved using either a (modified) Delphi process (n = 7)^42,44,47,49,50,62,65^ or RAND Corporation/University of California Los Angeles (UCLA) Appropriateness Method (n = 5).^41,48,51,54,63^ Outside of field testing (see below), there were no identified consistencies reported among these papers that used known consensus building methodologies – their contexts ranged from low- and middle-income countries to high income countries; sub-national, national and international indicator sets were all represented; and the dates of publication ranged from 2006 to 2020, similar to the papers that did not use the known consensus methodologies. In the cases that neither Delphi nor RAND/UCLA Appropriateness methods were explicitly applied, there were consultations reported with a carefully selected expert group or stakeholders relevant to the context.^38-40,43,45,46,52,53,56-59,61,64^ These accounted for half of the papers included (n = 14). Around one third of all included papers cited at least one patient representative as part of their consultations (n = 9).^40,43,45,47,48,50,51,54,56^

 While the methodology of this review explicitly states that field testing the derived indicators in practice is part of the inclusion criteria, this concept of testing in practice was not applied uniformly across each of the included indicator selection processes. Just over half of the included papers (n = 16) reported field testing where by adjustments could be made to the indicator set in response to the findings ie, there was utility in testing the indicators for the purpose of improving them and their application.^39-41,43,46-52,54,60,62,63,65^ All but two^42,44^ of the papers that reported using a known consensus methodology (Delphi or RAND/UCLA Appropriateness methods), conducted field testing in this way (n = 10).^41,47-51,54,62,63,65^ For those papers that did not adopt this approach to field testing, the indicator selection processes tested the indicators in practice more as a proof of concept and the opportunity to respond to feedback was not clear.

###  Structure of Frameworks and Indicator Criteria

 Of all included indicator selection processes, only one produced a single synthetic indicator^44^ and the remaining indicator sets were organised into frameworks (see Table 2). The frameworks were predominantly structured around PHC categories (n = 22).^38,40-43,45-51,53-60,63,64^ While there was some overlap of defined categories across included papers, these were limited. Of 123 categories of indicators identified, overlaps could be identified across nine categories: access, efficiency, effectiveness, safety, clinical care, quality, people, preventive health activities and chronic disease management. In contrast, a small selection of indicator selection processes used a causal chain structure, either Donabedian’s structure-process-outcomes classification (n = 2)^39,52^ or standard logic model (n = 3).^61,62,65^ Interestingly, these five papers did not include national level indicators (ie, they were either international or sub-national indicators), and four of them were published within the last four years.^39,52,62,65^

 In terms of indicator criteria, every included paper had considered the validity of each proposed indicator as part of their selection process. Feasibility issues, including consideration of data availability, reliability, integration with existing systems and/or whether a modification of existing practices would be required, also featured in all indicator selection processes but to varying degrees. In six of the included papers, this formed a central tenet of the selection process.^43,45,51,57,62,64^ Around half of papers factored in the resulting burden of reporting relative to context that can be generated by large and complex indicator frameworks (n = 13).^39,41-44,48,50,51,53,56,59,62,64^ The were no other consistent characteristics identified among these studies that considered reporting burden. The need for ongoing review was also reported inconsistently with just six of 28 included papers earmarking to revisit application of the indicator set in the future.^41,42,47,56,59,62^ Not all papers that reported ongoing review were included on the basis of a revision (see methodology), although half of them did (n = 3).^41,42,56^

###  Quality Assessment 

 We did not undertake a separate quality appraisal for individual studies as quality assessment of the processes used in each of the included papers formed the basis of our data extraction process and analyses. While our approach did not directly assess different aspects of indicator process against predefined benchmarks, as this is the aim beyond the findings of this paper, we did ascertain meaningful information on different aspects of indicator selection processes. For example, extracting data around formal consensus processes provided insights to the rigour applied to the study design. Likewise, extracting data around consulted stakeholders, including patient representatives, provided insights into the level of stakeholder engagement incorporated into the study design.

## Discussion

 Our review provides an overview of the key features of an indicator selection process (the process) used in the selection of PHC indicators. After a comprehensive search of databases and grey literature sources, 28 indicator selection processes met the inclusion criteria, where the indicators were subsequently field tested. We found no consistent variations between selection processes of health systems of high income and low- or lower-middle income countries.

 A literature review was the most common initial step, with adaptation of an existing framework also prevalent, but less common. A consensus building process with a range of stakeholders featured in nearly all of the included processes, with only around a third reporting inclusion of patient perspectives. A structured methodology for the consensus building process was not universally applied. Field testing was also only integrated into the process for just over half the papers. The resultant indicators were predominantly structured into PHC categories with limited overlap of categories across the different processes. All processes considered validity and feasibility issues while the reporting burden relative to resources was considered in around half of included papers and few papers reporting the need for ongoing review.

###  Fit for Use

 The term fit for use has been used to reflect that health system performance indicators, including those for PHC, are highly context specific and are needed by different parts of the health system, at different times. The selection of indicators should therefore aim to be well adapted to the context in which they are implemented while allowing assessment of a benchmarked standard of PHC. Currently there is no agreed criteria for assessment of fit for use for health system performance or PHC indicators. Recent work by Barbazza at el explored the complementary concepts of fit for purpose, fit for use and how these apply to the overall actionability of indicators.^12^ They identified three clusters for consideration when it comes to fit for use – methodological, contextual and managerial considerations.^12^ Determinations on whether an indicator selection process was successfully implemented in terms of meeting fit for use criteria was beyond the scope of this paper. However, future work could merge our findings with these categories to consider such an assessment.

 We sought to review the literature on selection processes of indicator sets that had already been implemented in practice and examining them through the lens of key criteria drawn from indicator appraisal frameworks.^10,11,36^ These appraisal frameworks were developed as assessment tools for health system performance indicators, with one of them designed specifically for assessing performance indicators for PHC.^36^

 A similar approach of using an indicator appraisal framework was adopted by de Bruin-Kooistra et al for selecting quality indicators for midwifery care in the Netherlands.^67^ The authors used the AIRE instrument as a manual and subsequent checklist for developing the indicators in their project.^67^ Likewise, Perara et al also incorporated a checklist into their development of the Systematic Indicator Development Method.^68^ However, they use a definition of “fitness for purpose” that gives little consideration to use beyond technical capacity.^68^

 One of the first papers to raise the notion of a ‘best practice’ selection process for health performance indicators was published in 2003 by Mainz.^69^ More recently, a systematic review by Kötter et al was undertaken analysing guideline based approaches to indicator development (including selection).^70^ The authors advocate for a ‘gold standard’ process of indicator selection to foster transparency and efficiency of resources and conclude that “It remains unclear which method leads to the best [*quality indicators*], since no randomized controlled or other comparative studies investigating this issue exist” (p. 20).^70^ Our systematic review adds to this groundwork and goes further to propose that any indicator selection process considered ‘gold standard’ will need to be sufficiently nimble to accommodate different contexts and therefore produce indicators that could be fit for use.

###  Consensus Among Stakeholders

 One of the emerging themes present in nearly all the processes was a procedure for reaching consensus among stakeholders. Not only were relevant stakeholders consulted but they were engaged in a deliberate way to reach consensus on the indicator set being developed. Two methodologies that feature strongly in the literature did not emerge as strong themes in our analysis: the Delphi technique and the RAND/UCLA appropriateness method. By definition, the Delphi technique involves repetitive administration of anonymous questionnaires, usually two or three rounds, with each round building towards a consensus usually without a face to face meeting.^71^ The RAND/UCLA appropriateness method, a derivative of the Delphi technique, similarly involves a series of rounds to reach consensus but adopts a more comprehensive approach by expressly combining expert opinion (from questionnaires and in person panel meetings) with evidence, usually drawn from a systematic literature review.^71^ Both of these methods have been used widely in the literature.^14-17,72,73^

 It is not clear why these or an alternative method did not feature more strongly among the selected papers in our review. Most papers that did use one of the consensus generating methodologies did also include field testing with utility (see below). As such, potentially it is an issue of resources. A recent systematic review by Jandhyala on consensus generating methods suggests that they have been modified over time and no longer reflect their original principles.^72^ In addition the inclusion of patients as part of stakeholder consultations was identified in only a third of the selected papers. This finding does not align well with the literature for best practice indicator selection as there is a large body of evidence that advocates for the inclusion of patients among stakeholder consultations.^21,74-79^

###  Utility of Field Testing

 One of the unique aspects of our review’s methodology is the inclusion of criterion around field testing. It was included to ensure implementation issues were adequately considered in line with the concept of selecting fit for use PHC indicators. There are many proposed indicator sets in the literature that are formed on the basis of a literature review, followed up with a consensus process among relevant experts. The goal of these papers is often to arrive at a final set indicators rather than ensure the indicator set is meeting the requirement that determined the need for the indicator(s) in the first place.^14-17,73,80^ In the absence of field testing, any proposed indicator(s) would still be theoretical.

 Our review identified that around half of the included papers incorporated a field testing component in a way that added utility to the indicators’ selection process.^41,43,46-52,57,62-65^ Other condition specific indicator selection processes in the literature have also emphasised this approach or the absence of it.^19,80-82^ Of note, Hilarion et al articulate that “…indicator development and their application should not be separated” (p. 99).^80^

###  Structure of Indicators

 Only few studies selected in our review structured their indicators according to a causal chain such as Donabedian’s structure-process-outcomes^83^ or a more standard logic model. Those that did not were structured around categories relevant to the context and there was limited overlap between the selected papers on PHC aspects. It is not clear why there was a preference for a non-linear categorisation of indicators over a sequential causal chain. Potentially a categorisation approach allows for easier comparison across different contexts and more flexibility to align within existing established frameworks.

 It is not clear which approach is favoured by the literature.^2,18,84^ For example, one widely used assessment tool for PHC, the Primary Care Assessment Tools, is organised according to principles of PHC – first contact, person-focused care over time, comprehensiveness and coordination.^30^ Further, on behalf of the World Organization of Family Doctors’ executive committee, Kidd et al argue that if PHC indicator(s) are too focussed on clinical conditions, they risk subsequent action favouring vertical oriented approaches.^85^ They suggest standard aspects of PHC be integrated into indicator frameworks such as comprehensiveness, coordination, continuity of care, safety and quality and workforce selection.^85^ However, grouping indicators according to Donabedian’s structure-process-outcome categorisation has also featured in the literature.^5,18^ A recent 2019 umbrella review of PHC quality indicators uses this grouping as a framework for analysing the indicators identified through its review.^18^

 In addition, our review only identified one selection process for a single synthetic indicator. This finding aligns with recent literature advising against composite indicators, in favour of multidimensional frameworks. This is because they can mask what is happening in reality and even if they do indicate an issue, it is difficult to unpack the system and other related factors that would have led to that finding.^5,86^

###  Strengths and Limitations

 Our review has several strengths. It was conducted in line with PRISMA guidelines and used a simplified search strategy to ensure it would comprehensively capture the range of contexts and terminology used when developing PHC indicators. This syntax was decided upon because it allowed for a large variation in terminology within the same topic, even though it increased screening burden and subsequent researcher bias (discussed below). The review sets the scene for further work on criteria that could be used to assess a ‘best practice’ in selecting indicators for PHC.

 Limitations of this review relate to the homogeneity of the types of studies that were included. Most of the included papers had a descriptive case study design. When a qualitative appraisal tool was applied (ie, Mixed Methods Appraisal Tool^87^), it yielded the same results for each included study because there was an overlap between the inclusion criteria and that of the appraisal tool. This meant appraising the quality of individual studies or assessing the risk of bias using an existing quality appraisal tool did not produce results that could meaningfully distinguish the quality of evidence from each of the different included papers. Other systematic reviews with process research questions have also not applied quality assessment tools.^21,88^ As explained by Caroll and Booth, quality appraisal in qualitative research is still vulnerable to subjectivity and any tool applied may only evaluate the reporting of the study rather than its actual conduct, thereby questioning its value.^89^ Further, the outcomes of this review are more closely aligned with methodologies for developing quality assessment tools. In the continuum proposed by Whiting et al, this review could be considered within the second stage of ‘tool development.’^90^ Thereby, attempting to apply an appraisal tool to this kind of tool development process research is duplicative and does not contribute to the credibility of the studies selected, as intend by quality appraisal.

 Process research is an emerging methodology in health systems research and is more commonly applied in psychotherapy and business management research.^88,91^ In the context of psychotherapy literature, process research is used to identify, describe, explain and predict the effects of processes that lead to therapeutic change and understand the mechanisms of action for a given result.^88^ There is more variation in how process research is applied in the field of business management. One application is in the context of New Venture Creation and focuses on the process of non-existence to existence of economic activities.^91^ Across these disciplines it is clear that there is value in understanding the process to achieve a given outcome, yet challenges remain in ensuring the rigour and absence of bias in qualitative process research. Berends and Deken argue that the challenge lies in demonstrating the link between process data and process theory.^92^ This is especially challenging when considering novel research questions where a clear theory is yet to be established, as is the case with this systematic review, which relied on pre-existing indicator appraisal tools as a foundation to understanding indicator selection processes. An exploratory or comparative design would offer more definitive insights about what features of an indicator selection process are conducive to fit for use PHC indicators. For example, an exploratory case study,^66^ qualitative comparative analysis^93^ or quasi experimental field trial^94^ with an emphasis on qualitative data collection, although these designs are more difficult and resource intensive. Also, most of the included papers were set in high-income countries which may affect the translation of the findings into other country contexts. However, the underlying theme of this analysis is to identify process features that transcend context so the impact of such differences on the findings may be limited. Further, the criteria applied to restrict included papers to PHC settings limits the generalisability of the findings, even though some characteristics may resonate at multiple levels of the health system.

 In addition, the base assumption for our findings is that comparisons across criteria drawn from a selection of health system indicator appraisal tools, among indicator sets that had been implemented in practice, leads to knowledge about the criteria for assessing the selection of indicators that are fit for use. In reality, there could be several factors in a given indicator selection process that could lead to indicators that are fit for use and were not captured by criteria used in our dataset. It is also possible that by restricting our criteria to indicators that were implemented in practice, comprehensive indicator selection processes in scope may have been excluded if such a process was developed and reported across more than one paper. The implementation criteria led to the exclusion of a number of papers during the title and abstract screening. Inclusion of these papers may have broadened the range of study designs included but this is unlikely as the research question would still focus on processes which are commonly reported through case study designs.

###  Researcher Bias

 Key aspects of the inclusion criteria were inherently vulnerable to researcher bias. For example, by including only papers that had sufficient information to extract for data collection, it is possible that there were papers that reported on indicator selection and were excluded due to the subjective level of detail in which they had described their processes. Further, by excluding less comprehensive or earlier papers when the same framework was reported across more than one paper, it is possible that different or more comprehensive processes were excluded, and may have contributed to the homogenous set of results.

 Our results may have also been affected by the variation of key words and naming conventions in this field which meant the selection of papers for inclusion were subject to researcher bias. The categorisation process undertaken to do data extraction and the subsequent analysis are other examples of unavoidable researcher bias.

 Lastly, a systematic review protocol was not registered for this study, limiting the transparency and opportunity for peer feedback on the methodology. The in-house protocol is available upon reasonable request from the corresponding author.

## Conclusion

 We identified several characteristics of health system indicator selection processes in the literature. These includeuse of a literature review as an initial step, more so than adapting an existing framework; stakeholder engagement with a known methodology to consensus building; structuring the indicator framework according to context specific PHC domains; and indicator criteria focusing on validity and feasibility (including reliability). The evidence around field testing with utility and consideration of reporting burden was not as strong despite being critical to implementation success. The evidence presented here provides some key principles to guide future work on assessing PHC indicator selection processes for health program staff, policy officials, donors and researchers. Future research using an explorative or comparative designs will strengthen these findings.

## Ethical issues

 Not applicable.

## Competing interests

 Authors declare that they have no competing interests.

## Authors’ contributions

 NR, AR, KL, and EF designed the study. NR completed the database and grey literature searches. Both NR and EF undertook the screening process and quality review. NR analysed the data and wrote the paper with input from all authors.

## Funding

 This research is supported by an Australian Government Research Training Program Fee Offset Scholarship and the Australian Government Research Training Program Domestic Scholarship.

## Supplementary files

Supplementary file 1. Detailed Search Strategy.
