# Supplementary material for: Common Features of Selection Processes of Health System Performance Indicators in Primary Healthcare: A Systematic Review
Source: Int J Health Policy Manag. 2022 Mar 17;11(12):2805–15. doi: 10.34172/ijhpm.2022.6239 (PMC10105193; doi:10.34172/ijhpm.2022.6239)
Supplement: Supplementary file 1 — Detailed Search Strategy. [file ijhpm-11-2805-s001.pdf]

**Article title:** Common Features of Selection Processes of Health System Performance Indicators in Primary Healthcare: A Systematic Review

**Journal name:** International Journal of Health Policy and Management (IJHPM)

**Authors' information:** Nicole Rendell[1]*, Alexander Rosewell[2], Kamalini Lokuge[1], Emma Field[1,3]

[1]National Centre for Epidemiology and Population Health, Australian National University, Canberra, ACT, Australia.

[2]School of Public Health and Community Medicine, University of New South Wales, Sydney, NSW, Australia.

[3]Menzies School of Health Research, Brisbane, QLD, Australia.

(*Corresponding author: nicole.rendell@anu.edu.au)

**Supplementary file 1.** Detailed Search Strategy

This supplementary document details the search strategy for the systematic review, *Common Features of Selection Processes of Health System Performance Indicators in Primary Health Care: A Systematic Review*.

**Search for indicator selection processes**

We aimed to use methods which were systematic, transparent, potentially replicable and aligned with the Preferred Reporting Items for Systematic Reviews and Meta-Analyses (PRISMA) guidelines[1].

An overview of the process is summarised below:

Step 1
- Define criteria for data extraction using literature on indicator quality (*outcomes*).

Step 2
- Run searches in each of the three databases.
- Remove duplicates.

Step 3
- Screening using Covidence* software. This included a title and abstract screening followed by a full text screening.

Step 4
- Review full text.
- Reach consenus on papers to be retained.

Step 5
- Quality appraisal of included papers (however we found no suitable tool to meaningfully complete this step).

Step 6
- Extract data into table based on criteria defined in step 1.

Step 7
- Analysis by grouping key themes emerging from table in step 6.

Step 8
- Synthesise findings.

*Covidence[2] is a digital tool to support management of screening papers in line with systematic review methodologies.

**Development of the search strategy**

The search strategy was developed in consultation with all members of the research team. This included:

- Identification of search terms and limits (or rather lack thereof) that respond to research question
- Identification of potential databases and grey literature platform to conduct searches
- Testing of the search strategy in Scopus and Medline
- Adjustment of the search terms to reduce the risk of bias when trying to define primary healthcare (PHC)
- Approval of the literature search strategy by the research team.

The lead author (NR) ran the searches in the Scopus and Medline databases and another author (EF) replicated the search in CINAHL. A global search using the Google platform in privacy mode was done to capture relevant grey literature. All results of the screening process were available in the Covidence[2] database set up for the review. Two authors (NR and EF) screened the titles and abstracts of the combined search results, identified documents of potential relevance, retrieved full versions of these papers and reports and conducted a second screen to identify relevant documents. NR completed the associated PRISMA flow diagram to report on the results at each stage.

Data were then extracted from the included documents and entered into the data extraction table. This table had been structured to incorporate relevant criteria drawn from published indicator appraisal tools that assessed the quality of health system performance indicators themselves, ie *outcomes*, to inform the structure of our dataset on indicator selection *processes*.[3-5]

**Overview of Research Question**

We aimed to review the literature to identify papers that document indicator selection processes for health system performance indicators in PHC.

**Search for:** Published papers or reports of indicator selection processes, for individual indicators or frameworks, that included PHC.

**Study type:** empirical studies or reports in the grey literature.

**Identification and definition of search terms:**

The search strategies were initially constructed, trialled, discussed, refined and then agreed by the research team.

**Databases searched:**

- Scopus
- Medline
- CINAHL

- Google global search.

**Time:** No time limits were applied.

**Population:** No population limits were applied but only documents with their full text available in English were included.

**Inclusions:**
- Indicator selection processes including any indicator or indicator set, that was identified for implementation and ongoing PHC management in a real world setting. These included clinical indicator series covering more than one disease and are used with the goal of understanding PHC performance.
- The care setting was considered in scope, if it aligned with the definition of PHC outlined by WHO[6] and no referral was required by an individual to seek the services.
- Indicators were field tested, piloted or implemented (field testing). This was interpreted to include revisions of an existing indicator set and those of well-established organisations known to the authors with a clear trajectory for implementation, of indicators yet to be clearly implemented. This criterion was to ensure practical considerations of implementation were captured by the selected studies.

**Exclusions:**
- Empirical studies that reported only on indicators related to hospitals or acute settings
- Empirical studies that developed and/or applied only a survey design without consideration for selection of PHC indicators
- Secondary sources (for example, narrative reviews and systematic reviews)
- Indicators specific to a single condition, due to our focus on health system performance
- Indicators based on a theoretical discussion on health system performance assessment including proposed indicators or frameworks that had not been field tested.

**Comparator:** Indicator quality criteria drawn from published indicator appraisal tools.[3-5]

**Outcomes:** Common features of selection processes of health system performance indicators in PHC.

**Syntax by Database**
No limits were applied for any of the searches below
- Scopus
    - Article Title, Abstract, Keywords search
      ("health system?" OR "health care" OR "primary health*" OR "primary care") AND ("performance indicator?" OR "quality indicator?" OR "framework?") AND (development OR prioriti?ation OR selection) AND NOT (acute OR hospital).
- Medline

    - Multi field search, all fields
      ("health system?" or "health care" or "primary health*" or "primary care") and ("performance indicator?" or "quality indicator?" or "framework?") and (development or prioriti?ation or selection)) not (acute or hospital)
- CINAHL
    - Boolean/Phrase mode
      ("health system?" OR "health care" OR "primary health*" OR "primary care") AND ("performance indicator?" OR "quality indicator?" OR "framework?") AND (development OR prioriti?ation OR selection) **AND** NOT (acute OR hospital)

**Reference List**


1. Moher D, Liberati A, Tetzlaff J, Altman DG, The PG. Preferred Reporting Items for Systematic Reviews and Meta-Analyses: The PRISMA Statement. *PLOS Medicine.* 2009;6(7):e1000097. doi:10.1371/journal.pmed.1000097
2. Covidence. Better systematic review management. nd. https://www.covidence.org/.
3. Perera R, Dowell T, Crampton P, Kearns R. Panning for gold: An evidence-based tool for assessment of performance indicators in primary health care. *Health Policy.* 2007;80:314-327. doi:10.1016/j.healthpol.2006.03.011
4. de Koning J, Smulders A, Klazinga N. Appraisal of Indicators through Research and Evaluation (AIRE). Amsterdam: Academic Medical Center- University of Amsterdam; 2007.
5. Reiter A, Fischer B, Kötting J, et al. QUALIFY: Instrument for the Assessment of Quality Indicators. 2007. https://www.researchgate.net/publication/267256474_QUALIFY_Instrument_for_the_Assessment_of_Quality_Indicators.
6. World Health Organization. Primary Health Care (PHC). 2019; https://www.who.int/primary-health/en/.
